# To buy or not to buy—evaluating commercial AI solutions in radiology (the ECLAIR guidelines)

**DOI:** 10.1007/s00330-020-07684-x

**Published:** 2021-03-05

**Authors:** Patrick Omoumi, Alexis Ducarouge, Antoine Tournier, Hugh Harvey, Charles E. Kahn, Fanny Louvet-de Verchère, Daniel Pinto Dos Santos, Tobias Kober, Jonas Richiardi

**Affiliations:** 1 Department of Radiology, Lausanne University Hospital and University of Lausanne, Rue du Bugnon 46, 1011 Lausanne, Switzerland; 2 Gleamer, Paris, France; 3 Hardian Health, Haywards Heath, UK; 4 Department of Radiology, University of Pennsylvania, Philadelphia, PA USA; 5 IBM Watson Health, Paris, France; 6 Department of Radiology, University Hospital of Cologne, Cologne, Germany; 7 Advanced Clinical Imaging Technology, Siemens Healthcare AG, Lausanne, Switzerland

**Keywords:** Artificial intelligence, Software, Legislation, Workload, Equipment and supplies

## Abstract

**Abstract:**

Artificial intelligence (AI) has made impressive progress over the past few years, including many applications in medical imaging. Numerous commercial solutions based on AI techniques are now available for sale, forcing radiology practices to learn how to properly assess these tools. While several guidelines describing good practices for conducting and reporting AI-based research in medicine and radiology have been published, fewer efforts have focused on recommendations addressing the key questions to consider when critically assessing AI solutions before purchase. Commercial AI solutions are typically complicated software products, for the evaluation of which many factors are to be considered. In this work, authors from academia and industry have joined efforts to propose a practical framework that will help stakeholders evaluate commercial AI solutions in radiology (the ECLAIR guidelines) and reach an informed decision. Topics to consider in the evaluation include the relevance of the solution from the point of view of each stakeholder, issues regarding performance and validation, usability and integration, regulatory and legal aspects, and financial and support services.

**Key Points:**

*• Numerous commercial solutions based on artificial intelligence techniques are now available for sale, and radiology practices have to learn how to properly assess these tools.*

*• We propose a framework focusing on practical points to consider when assessing an AI solution in medical imaging, allowing all stakeholders to conduct relevant discussions with manufacturers and reach an informed decision as to whether to purchase an AI commercial solution for imaging applications.*

*• Topics to consider in the evaluation include the relevance of the solution from the point of view of each stakeholder, issues regarding performance and validation, usability and integration, regulatory and legal aspects, and financial and support services.*

**Supplementary Information:**

The online version contains supplementary material available at 10.1007/s00330-020-07684-x.

## Introduction

Artificial intelligence (AI) has made impressive progress over the past few years, fueled in large part by advances in its subfield of deep learning (DL). DL, itself part of machine learning (ML), is the main focus of these guidelines and corresponds to a class of algorithms that learn directly from data to produce the desired output, and has posted human-level (or superhuman) performance in tasks such as image recognition. This enthusiasm has spilled over into medical imaging, and radiology departments are increasingly receptive to such techniques, which promise to help cope with ever-expanding workloads and ultimately improve patients’ outcome. To help radiologists and other physicians find their way in this new world, several guidelines describing good practices for conducting and reporting AI-based research in medicine and radiology have been published [1–6].

This rapid academic progress has been paralleled by unprecedented investment and activity in private and public companies, with numerous commercial solutions based on AI techniques now available for sale. Medical device regulation has also evolved, in particular with the notion of AI-based software as a medical device (SaMD) [7]. Evaluation of AI offerings must include technical and financial considerations, quality and safety factors, and input from key stakeholders [8, 9]. Thus, our main goal is to bring together authors from academia and industry in order to provide a guide that will help radiologists select the most appropriate commercial AI solution for their needs, through a set of questions to challenge solution providers when evaluating commercial AI solutions in radiology (the ECLAIR guidelines). These are summarized in Table 1, and Table 2 highlights the top 10 questions to consider. In the remainder of this paper, we will assume that the reader has a basic knowledge of AI and AI-related terminology, which can be found in other publications aiming at introducing AI to radiologists [10–13]. We also provide a glossary as supplementary material for reference.

**Table 1:** Checklist of points to consider when assessing a commercial AI solution in radiology

1. Relevance	**1.1. What problem is the application intended to solve, and who is the application designed for?** *Define the scope of application; end-users; research vs. clinical use; usage as double reader, triage, other; outputs (diagnosis, prognosis, quantitative data, other), indications and contra-indications*
	**1.2 .What are the potential benefits, and for whom?** *Consider benefits for patients, radiologists/referring clinicians, institution, society*
	**1.3. What are the risks associated with the use of the AI system?** *Consider risks of misdiagnosis (including legal costs), of negative impact on workflow, of negative impact on quality of training*
2. Performance and validation	**2.1. Are the algorithm’s design specifications clear?** *Check robustness to variability of acquisition parameters; identify features (radiomics) or network architecture (deep learning) used*
	**2.2. How was the algorithm trained?** *Assess population characteristics and acquisition techniques used, labeling process, confounding factors, and operating point selection*
	**2.3. How has performance been evaluated?** *Check proper partitioning of training/validation/testing data, representativeness and open availability of data. Assess human benchmarks, application scope during evaluation, source of clinical validation*
	**2.4. Have the developers identified and accounted for potential sources of bias in their algorithm?** *Assess training data collection, bias evaluation, stratification analyses*
	**2.5. Is the algorithm fixed or adapting as new data comes in?** Check whether user feedback is incorporated, if regulatory approval is maintained, and if results are comparable with previous versions. *
3. Usability and integration	**3.1. How can the application be integrated into your clinical workflow?** *Consider integration with your information technology (IT) platform, check for compliance with ISO usability standards, consider issues related to practical management of the software*
	**3.2. How exactly does the application impact the workflow?** *Identify modifications to bring to your current workflow, identify roles in the new workflow (physicians and non-physicians)*
	**3.3. What are the requirements in terms of information technology (IT) infrastructure?** *Consider on-premise vs. cloud solutions. Identify requirements in terms of hardware and network performance, consider network security issues*
	**3.4. Interoperability - How can the data be exported for research and other purposes?** *Check whether the export formats are suitable*
	**3.5. Will the data be accessible to non-radiologists (referring physicians, patients)?** *Check whether the form of the output is suitable for communication with patients/referring physicians*
	**3.6. Are the AI model’s results interpretable?** *Check whether and which interpretability tools (i.e. visualization) are used*
4. Regulatory and legal aspects	**4.1. Does the AI application comply with the local medical device regulations?** *Check whether the manufacturer obtained regulatory approval from the country where the application will be used (CE, FDA, UKCA, MDSAP, or other local guidance), and for which risk class*
	**4.2. Does the AI application comply with the data protection regulations?** *Check whether the manufacturer complies with local data protection regulations and provides contractual clauses protecting patient’s data*
5. Financial and support services considerations	**5.1. What is the licensing model?** *Assess one-time fee vs. subscription models, total costs, scalability*
	**5.2. How are user training and follow-up handled?** *Check whether training sessions are included and at which conditions further training can be obtained*
	**5.3. How is the maintenance of the product ensured?** *Check whether regular maintenance is included, assess the procedure during downtime and for repair*
	**5.4. How will potential malfunctions or erroneous results be handled?** *Assess the procedure in the event of malfunction and post market surveillance and follow-up*

* Note that at the time of writing of these guidelines, no adaptative AI application exists on the market.

**Table 2:** Top 10 questions to consider

1. What problem is the application intended to solve, and who is the application designed for?	
2. What are the potential benefits and risks, and for whom?	
3. Has the algorithm been rigorously and independently validated?	
4. How can the application be integrated into your clinical workflow and is the solution interoperable with your existing software?	
5. What are the IT infrastructure requirements?	
6. Does the application conform to the medical device and the personal data protection regulations of the target country, and what class of regulation does it conform to?	
7. Have return on investment (RoI) analyses been performed?	
8. How is the maintenance of the product ensured?	
9. How are user training and follow-up handled?	
10. How will potential malfunctions or erroneous results be handled?	

## Relevance

### What problem is the application intended to solve, and who is the application designed for?

In assessing the relevance of the AI solution to one’s practice, the fundamental questions to answer are what specific problem it is supposed to solve (the intended use), and under what conditions (the indications of use)? There should be a clear specific clinical indication (the use case) that the vendor should be able to explain.

Basic points to consider include:

- What are the medical conditions to be diagnosed, treated, and/or monitored?
- Who are the intended end-users—i.e., radiologists, clinicians, surgeons—as well as their required qualifications and training?
- What are the principles of operation of the device and its mode of action?
- Is the application intended to be used as a research tool or for clinical use?
- Will the AI solution be used as a double reader, to triage examinations, to perform quality control, or for some other function [10]?
- Does the system produce a diagnosis, a prognosis, or quantitative data (lesion segmentation, organ volumes, etc.)? [14, 15]
- Does the application provide useful information that was not available before?
- Are there any other considerations such as patient selection criteria, indications, contra-indications, warnings?
  For SaMD, the “intended use statement” of the product regulatory documentation should provide this information.

### What are the potential benefits, and for whom?

Benefits can be assessed from the perspective of patients, radiologists, referring physicians, hospitals, insurance companies, the healthcare system, or society as a whole. Each view has its own outcome measures, some of which are reported below. Ideally, benefits should be linked to evidence, including scientific publications and healthcare economics analyses.

#### Patients

AI software may increase the value of imaging in patient care in many ways [16]. Outcome measures to assess the impact on patient management, such as diagnostic performance, diagnostic impact, therapeutic impact, and quality of life, are detailed in dedicated publications [17].

#### Radiologists and referring physicians

Some of the benefits and outcome measures to consider from the perspective of radiologists and clinicians include:

- Increased productivity and decreased reporting time, which can impact clinician’s and radiologist’s satisfaction [18]
- Increased time spent with patients, which can impact patient’s and radiologist’s satisfaction [19]
- Reduced time spent on “menial” tasks
- Faster diagnosis in time-sensitive situations (e.g., stroke)
- Potential decrease in physical or psychological strain
- Increased quality control, reduced malpractice risk, legal and insurance costs

#### Institution

Potential benefits for the institution include improved physician efficiency, more effective resource utilization, more rapid care processes, and reduced malpractice risk. Formal health economics assessments, such as cost-benefit and cost-effectiveness analyses, are scarce and should be encouraged. Solution vendors could be a good source of return on investment (RoI) analyses; although they are likely to be optimistic, they should at least provide suitable RoI metrics that can be recorded and confronted with reality as the system is deployed. Different health care systems will require different health economic modeling to ensure local RoI viability.

#### Society

Potential societal benefits include decreased healthcare costs, increased access to healthcare with decreased variability in the quality of care, and, ultimately, increased life expectancy and quality of life.

### What are the risks associated with the use of the AI system?

All the benefits above come with related risks. In general, the buyer should ask to review the risk assessment matrix and risk-benefit analysis in the regulatory technical file provided by the vendor, which covers some of these risks.

Risks related to the use of AI solutions, such as misdiagnosis, generate legal exposure. In this regard, the risks for the buyer’s institution should be identified, and responsibilities clearly assigned.

Other risks must be considered. The radiologist’s workflow could be impacted negatively in case of poor integration (see section “Usability and integration”) or poor reliability of the AI system. Furthermore, although the training of radiologists might be improved by an always-available double reader, AI could have a negative impact by causing trainees to rely too much on it, or more importantly, to neglect basic knowledge of imaging signs.

## Performance and validation

As for any diagnostic solutions, AI algorithms need to be assessed following the standards for unbiased assessment in a clinical context (e.g., STARD, TRIPOD, CHARMS), and diagnostic performance measures must be available [3, 20, 21]. Nevertheless, AI-based products have specific features that require particular guidance (e.g., TRIPOD-ML, CONSORT-AI, SPIRIT-AI, and CLAIM guidelines) [1–6].

### Are the algorithm’s design specifications clear?

Small details can have cascading effects on the performance of AI algorithms [22, 23]. Thus, AI software vendors need to disclose many details about how their software operates in order to explain how real-world clinical imaging data can be accommodated. Typically, a design specification should be included in the technical file. In particular, vendors should explain:

- Which image processing steps are used? How are differences in resolution, contrast, and intensity handled on images from different machines?
- For radiomics approaches, which features does the algorithm assess? How does the algorithm represent images prior to learning and analysis? This information can then be linked back to peer-reviewed literature for critical appraisal of performance.
- For deep learning AI algorithms, which neural network is used (e.g., U-Net is a popular architecture for segmentation)? Such information, ideally with reference to the relevant literature, may help identify possible failure modes of the algorithm. Vendors should be able and willing to explain broadly how their algorithms operate to both non-specialists and specialists embedded within radiology departments. If not, this should count as a negative point in the competitive analysis with other solutions.

### How was the algorithm trained?

AI algorithms include many parameters, which must be learned or “trained” from data (medical images) and labels—annotations, which can be as broad as a diagnosis attached to a whole image, or as specific as labeling particular voxels with tags such as “lesion” or “necrosis.” They are then validated on separate data (possibly multiple times) and finally should be tested on external data, from another cohort or machine. This last point is particularly important as it guards against overfitting. Thus, ultimate performance depends critically on the data used. In general, one may refer to detailed guidelines [3, 21, 24], but several points are of particular importance:

- What data was used to train the AI algorithm? This must include the number of patients, controls, images, and occurrence of pathology or abnormality. Clinical and demographic data on patients (with inclusion and exclusion criteria) must be provided, together with information about location and type of acquisition sites. Technical parameters including vendors, modalities, spatial and temporal resolution of images, acquisition sequence details, field strength if applicable, patient position, injection of contrast agents, and the like must be specified. The sample used to develop the algorithms should have characteristics that are representative of the target population for which the algorithm will be used to avoid bias (i.e., same age, ethnicity, breast typology….), but also follow the same processing steps that will be applied during deployment [25].
- How was labeling performed? What was the experience level of readers? How many readers per case? Were the readers given realistic conditions for image interpretation? In particular, did they have access to native resolution images, with their usual viewers and tools? Did they have access to relevant clinical information and other images? Was there a time constraint?
- Are there confounding factors in the data? For example, in multi-site data, were more patients at one site diagnosed with a particular disease than in another site?
- Based on which criteria were the operating points chosen, and on which dataset?

### How has performance been evaluated?

First, for proper evaluation of generalizability, all algorithms should be developed and evaluated on *disjoint subsets of the dataset*. This essentially means that the algorithm should not be tested on the same data on which it was developed. The TRIPOD guidelines show different approaches to achieve this. Some questions are common to all types of algorithms:

- What data was used to validate and tune the AI algorithm? Is there an overlap with the training data? If so, this is a red flag.
- What data was used to test the AI algorithm? Is there an overlap with the training and validation data? Again, this is a red flag.
- Is the test set realistic? Is it representative of the population in which the system will be used (e.g., age, sex, BMI, prevalence of pathologies, comorbidities)? If not, radiologists should be aware that results could be sub-optimal in some cases that have not been thoroughly tested, such as obese patients.
- Are the test set (including imaging and clinical data), and the ground truth available and/or open for reproducibility?
- Has the algorithm been benchmarked against experts in the field?
- Are performance results reported for the AI algorithm as a stand-alone clinical decision support system, or as a second reader? Has the added value for human readers (in terms of performance) been assessed?
- Is the clinical validation done by sources external/independent from the creator of the algorithm? Is the clinical study design of good quality?

For practical use, it is particularly important to gauge how *robust* the algorithm is to technical variations in the images. The main points to assess are repeatability (same machine, same time (e.g., back-to-back acquisitions) and reproducibility (different machine, different sequence or contrast, or different time). These should be covered in the technical file. In particular, questions to consider include:

- How reproducible is the algorithm against variability in acquisition parameters (e.g. contrast, signal-to-noise, resolution parameters)? This is typically a weak point in academic/research systems, where AI algorithms can easily latch onto acquisition details unrelated to pathology if these are confounders, but commercial systems should present evidence that they are reproducible in the deployment environment [26].
- How repeatable (deterministic) is the algorithm? For algorithms outputting single values (e.g., volumetry), the repeatability coefficient and Bland-Altman plots should be provided.
- How does the algorithm handle differences in data quality? Was the algorithm evaluated on artefactual/non-ideal data? What were the results?

The performance metrics to be used depend on the type of algorithm and are detailed in existing guidelines [20, 21]:

- For *classification algorithms* (e.g., diagnosis): Are both threshold-dependent (e.g., sensitivity, specificity) and threshold-independent metrics (such as the area under the receiver operating curve (ROC)) reported? For imbalanced datasets, are appropriate metrics (balanced accuracy, no-information rate, Kappa…) provided? Are confidence intervals provided?
- For *regression algorithms* (e.g., linking clinical scores or liquid biomarker levels to images, such as bone age assessment): Are both metrics of typical performance (mean average error (MAE)) and more extreme performance (root mean-squared error (RMSE)) provided? For forecasting (prognosis), is a benchmark with respect to the one-step naïve forecast, e.g., using mean absolute scaled error [27] (MASE), provided?
- For *detection algorithms* (e.g., anomaly detection in mammography): Are metrics presented both in terms of patient-level classification metrics with an explicit and motivated definition of true positive and negative, false positive and negative; and in terms of the trade-off between anomaly-level sensitivity and individual false positives rate, such as the free-recall ROC (FROC) curve? Is the matching criterion, such as intersection-over-union threshold, clearly defined?
- For *segmentation algorithms*: Are both overall voxel-level metrics such as Jaccard or Dice coefficients and absolute volume differences provided? Are instance-level metrics such as per-lesion accuracy metrics provided?

### Have the developers identified and accounted for potential sources of bias in their algorithm?

AI algorithms can learn human biases (e.g., towards race, gender, or socioeconomic status) from their training data or in their application. Awareness of the potential for bias is critical. Thus, vendors should be ready to discuss how their training data were collected, how the model was trained, and how the evaluation process ensures that outputs are as unbiased as possible [28]. Vendors should also be asked to provide evidence of hidden stratification or sub-stratification analyses to check for unknown biases affecting data sub-groups.

### Is the algorithm fixed or adapting as new data comes in?

AI algorithms typically are trained with a fixed dataset before being deployed. A more recent trend is to allow AI algorithms to continuously adapt by including more data, hereby improving performance and adapting to slow changes in imaging equipment and population. Relevant questions include:

- Does the system adapt to your local data over time or via updates?
- Is feedback obtained from the users (such as pointing out erroneous detections) incorporated?
- If the algorithm undergoes continuous improvement, is that covered by the regulatory approval? Currently, no adaptive AI systems are regulatory approved, though this may change as the technology progresses.
- If performance is increased in future updates, the algorithm is changed. How are results obtained with the prior versions handled? Will they still be valid and can one still compare them to the results obtained with the new version of the algorithm?

## Usability and integration

### How can the application be integrated into your clinical workflow?

Ideally, the data processing should take place in the background and be fast enough for the results to be available when the radiologist is reading and reporting examinations. Questions to consider include:

- Is manual interaction needed, or is the processing performed automatically in the background?
- How fast is the processing cycle from data acquisition to the result?
- How can the processing status of a specific dataset be checked?

The application to be used by radiologists should aim to be fully integrated with the picture archiving and communication system (PACS) and accessible with a minimum of mouse clicks—this is key for clinical usability. Are the AI tool and its results readily accessible in the working environment, and with a user-friendly interface? For SaMDs, it is important to check whether the vendor has undertaken validation according to the International Organization for Standardization (ISO) standards; ISO 62633 relates to the usability and safety of medical devices [29]. It is advisable to involve the IT department and PACS specialists early in the evaluation process.

Finally, questions to consider in terms of how the user can manage the software include:

- Is there integration of identity management with the hospital system?
- Are there different roles/users defined in the product?
- Who can assign new users and/or roles? How much work does this represent?
- If interaction is needed, are all actions trackable?

### How exactly does the application impact the workflow?

AI applications may be able to improve workflow. For example, triage and prioritization of the report list based on automatic identification of abnormalities can prioritize important cases. The application output such as qualitative or quantitative data could be used to automatically populate structured reports; impact on reading and reporting time should be quantified. However, it is important to identify all roles involved in the new workflow, including non-physicians such as technicians. Indeed, some resources might have to be reallocated for certain tasks. The reporting structure might also have to be changed with the use of the AI solution. These factors need to be taken into account in the decision process.

### What are the requirements in terms of information technology (IT) infrastructure?

For on-premise deployment, some AI applications may require specialized computer hardware such as graphics processing units (GPU), which are not present in all computers. Not all GPUs are equivalent, so requirements in terms of GPU computation power (e.g., “compute unified device architecture (CUDA) compute level”) and memory (e.g., “11 GB or more”) should be made clear. Some models cost significantly more than a typical desktop computer. Likewise, central processing unit (CPU) specifications, memory, disk storage, and energy requirements must be made clear. Depending on the above, the solution may require significant additional expenses if new hardware has to be acquired.

For cloud solutions, requirements are usually lighter. Nevertheless, network security and network performance issues need to be discussed. It is highly advisable to consult the IT department early.

### Interoperability—how can the data be exported for research and other purposes?

The output format and the accessibility of the results may impact interoperability. For applications that are not fully integrated in the PACS, it is important to consider the following questions:

- How can the data be exported for research purposes? Are there accessible application programming interfaces (API) such as a DICOMweb interface?
- Is the output in a standards-compliant format such as Digital Imaging and Communications in Medicine (DICOM) structured report (SR) following SR template identifier (TID) 1500?
- Are standard export formats (e.g., simple comma-separated values (CSV) format) supported?
- Are the results saved, or must the computation be performed anew every time?

Of note, some initiatives to improve interoperability of AI solutions with existing standards-based healthcare systems exist [30].

### Will the data be accessible to non-radiologists (referring physicians, patients)?

If applicable, consider whether the data are presented in a form that is suitable to be transferred to patients, or understandable by referring physicians.

### Are the AI model’s results interpretable?

Depending on how it is to be used, it may be critical for the AI system to be able to explain its reasoning or to provide a means for physicians to interpret its output. One popular approach is a visualization, where a heat map of the importance of specific image regions is overlaid on top of the initial image [31].

## Regulatory and legal aspects

Regulatory and legal requirements vary around the world. Nevertheless, due to their characteristics, AI applications used in radiology must comply with two main regulatory and legal frameworks: the medical device and the personal data protection regulations.

### Does the AI application comply with the local medical device regulations?

The manufacturer must define a use case for its application and specify whether it should be used as a medical device, and under which risk class. Medical devices are classified into risk classes from I to III; the riskier the medical device, the more regulatory controls are applied. Implementation processes vary from country to country and the first question that must be addressed is whether the AI application has been cleared/approved in the target country [32].

#### *For Europe, is the AI application CE marked?*

For class I medical devices, the manufacturer can perform self-certification and certify that its device is compliant with regulations, without the involvement of an independent body.

For higher risk classes, which represent the vast majority of AI-based SaMDs, the manufacturer must appoint a notified body that will review both the technical documentation of the medical device and the processes in place in the company before issuing a CE certificate.

In Europe, the regulatory framework to put a device on the market is currently changing from the Directive to the Medical Device Regulation (MDR) [33, 34]. The application of the MDR will enter into application on May 26, 2021. Medical devices that comply with the Directive are given a transition period of up to four years during which they can remain on the market. However, this is only applicable when no substantial modifications are planned on the medical device. Otherwise, the manufacturer would have to conform to the MDR. An additional question would then be is the manufacturer already planning on transitioning to the MDR?

From January 1, 2021, the United Kingdom (UK) will require international importers to register separately with the UK Medicines and Healthcare products Regulatory Agency (MHRA), while a transition from CE marking to UKCA takes place until the 30^th^ of June 2023 [35].

#### *For the US, is the AI application FDA-cleared or FDA-approved?*

To be lawfully put on the US market, a medical device must be reviewed by the FDA [36, 37], using either the De Novo pathway, for innovative medical devices that have no equivalent (FDA approval) [38]; or the 510(k) pathway, for medical devices that have an equivalent predicate already on the US market (FDA clearance) [39].

FDA approval/clearance is often considered a quality stamp because the FDA remains one of the most demanding regulators in the world. Thus, manufacturers might want to purposely reduce the scope of the AI application for the FDA submission file. Particular attention must be paid to the scope of an FDA-approved/FDA-cleared device, and whether the non-US version of the device is different from the US version, and how. Additionnally, the FDA is currently building a new regulatory framework for the evlaluation of AI-based SaMDs [40].

#### *Other medical device regulations*

For other geographical areas, there are three scenarios for regulatory approval.

First, the target country recognizes FDA clearance/approval and CE marking as equivalent to its level of requirements: in this case, the manufacturer faces less challenges but still must register with the local authorities.

Second, the target country does not recognize either FDA approval/clearance or CE marking and has its own regulation. It is the case for example in Japan where the manufacturer must submit an application to the Pharmaceutical and Medical Devices Agency (PMDA) or in China where the manufacturer must submit an application to the National Medical Products Administration (NMPA).

Third, several countries including the USA, Australia, Brazil, Canada, and Japan accept the Medical Device Single Audit Program (MDSAP) certification which is well-aligned with the MDR [41].

### Does the AI application comply with the data protection regulations?

AI systems handle sensitive health-related data that fall under regulations such as those in place for medical devices. For example, the General Data Protection Regulation (GDPR) is in place in Europe while the Health Insurance Portability and Accountability Act (HIPAA) applies to the US [34, 42].

Compliance with these regulations is most of the time a two-sided process with contractual arrangements between users and the manufacturer and security measures that need to be in place. The following questions can help assess the readiness of the manufacturer:

- What are the contractual guarantees given by the manufacturer? Are there specific clauses in the contract related to the protection of data?
- Does the manufacturer have a reference person for data protection issues?
- Does the processing of data occur on premise or remotely? Is the manufacturer or the subcontractor hosting the processing compliant with information security standards ISO 27001/27017/27018?
- Is the data pseudonymized, and if yes, where are the mapping tables stored?

## Financial and support services considerations

AI applications are complicated pieces of software that rely on several other software and hardware components. Thus, in addition to pricing, questions about maintenance, training, and support need to be discussed prior to committing to a purchase. This is in addition to the internal hospital IT costs mentioned in the “How exactly does the application impact the workflow” section.

### What is the licensing model?

With the rise of software-as-a-service (SaaS) and subscription models, it is not always trivial to compute total cost of ownership. The following questions must be clarified:

What is the business model: is it a one-time fee, a subscription, or a pay-per-use model? Are there discounts based on processed imaging volume? If it is a subscription plan, what are the cancellation procedure/delays?

- Does the manufacturer offer a trial period? Is it possible to proceed to a real-life evaluation of the product on the hospital’s own data before purchase?
- What are the exact costs now, and in the future (install costs, yearly software license, maintenance fees, costs of potential future updates, internal efforts, etc.)?
- How does the solution scale to more users, or more DICOM modalities (devices)? Would there be additional costs?
- Is the AI system offered through an “App store” portal from an established EHR, dictation, or PACS vendor, or AI marketplace? If so, will the purchase of that application simplify your access to other applications in the future (by leveraging the same computing architecture and/or AI user interface)?

### How are user training and follow-up handled?

Like other IT products, AI systems need some time to get used to and offer various degrees of user-friendliness. To ensure radiologists will use the system efficiently, training and support are necessary. The following points should be discussed:

- Does the purchase of the product include training sessions? Who should participate and how much time is required per function?
- Can additional training sessions be arranged for new users? How much would that cost?
- If a question comes up, is there a way to contact the vendor and a guaranteed reaction time?

### How is the maintenance of the product ensured?

Because AI systems live within a constantly evolving clinical ecosystem, questions around the maintenance of the product are important to consider. A few essential questions should clarify how that will happen:

- Will there be regular maintenance?
- If the product is down, would it still be possible to proceed with reading the relevant images by other means? What is the procedure for repair? What would be the delay? Who would have to cover the costs?
- What is the guaranteed uptime of the servers the software runs on?

### How will potential malfunctions or erroneous results be handled?

No AI application is perfect. With exposure to real-life, highly variable clinical datasets, errors can happen. The following points have to be clarified before purchase:

- How will malfunctions be addressed? If severe, is there a guarantee that the problem will be fixed?
- What is the pathway to file a potential malfunction? Is there an automatic monitoring in place or do the users have to report malfunctions?
- What are the adverse event reporting pathways?
- How is post market surveillance and post market clinical follow-up to be conducted?

## Conclusion

This work aims to provide a list of practical points to address when considering whether to invest in an AI solution in medical imaging. Although some assessment criteria presented here may not apply to every situation, we hope to have developed a framework that will allow all stakeholders to conduct relevant discussions with manufacturers and reach an informed decision.

## Supplementary material

ESM 1 (DOCX 22.7 kb)

## Abbreviations

AI: Artificial intelligence
API: Application Programming Interface
CE: Conformité Européenne
CPU: Central processing unit
CSV: Comma-separated values
CUDA: Compute unified device architecture
DICOM: Digital Imaging and Communications in Medicine
DL: Deep learning
FDA: Food and Drug Administration
FROC: Free-recall receiver operating characteristic
GDPR: General Data Protection Regulation
GPU: Graphics processing unit
HIPAA: Health Insurance Portability and Accountability Act
ISO: International Organization for Standardization
IT: Information technology
MAE: Mean average error
MASE: Mean absolute scaled error
MDR: Medical Device Regulation
MDSAP: Medical Device Single Audit Program
MHRA: Medicines and Healthcare products Regulatory Agency
NMPA: National Medical Products Administration
PACS: Picture Archiving and Communication System
PMDA: Pharmaceutical and Medical Devices Agency
RMSE: Root mean-squared error
ROC: Receiver operating characteristic
RoI: Return on investment
SaMD: Software as a medical device
SR: Structured report
TID: Template IDentifier
UK: United Kingdom
UKCA: United Kingdom conformity assessed
US: United States (of America)
