# Supplementary material for: To buy or not to buy—evaluating commercial AI solutions in radiology (the ECLAIR guidelines)
Source: Eur Radiol. 2021 Mar 5;31(6):3786–96. doi: 10.1007/s00330-020-07684-x (PMC8128726; doi:10.1007/s00330-020-07684-x)
Supplement: Supplementary file 1 — (DOCX 22.7 kb) [file 330_2020_7684_MOESM1_ESM.docx]

**Supplementary material: Glossary**

**510(k):** A 510(k) is a premarket submission made to the FDA to demonstrate that the device to be marketed is as safe and effective, that is, substantially equivalent, to a legally marketed device (section 513(i)(1)(A) FD&C Act). Submitters must compare their device to one or more similar legally marketed devices and make and support their substantial equivalence claims. [Premarket Notification 510(k). Available at: https://www.fda.gov/medical-devices/premarket-submissions/premarket-notification-510k. Accessed October 3, 2020] 

**Adaptive AI systems**: Refers to AI systems where the application continuously adapts by including more data, hereby improving performance and adapting to slow changes in imaging equipment and population. 

**Class I/IIa/IIb/III Medical Device:** In Europe, all medical devices are placed into one of four graduated categories, using the classification rules listed in Directive 93/42/EEC Annex IX or Regulation 2017/745 Annex VIII. The categories are Class I (including Is & Im), Class IIa and IIb and Class III, with Class III ranked as the highest. In the US, the FDA uses only classifications I,II and III. The higher the classification the greater the level of assessment required by Notified Bodies. It is the intended purpose of the device that determines the classification and not the particular technical characteristics. Considerations for classification include the duration of contact with the body, degree of invasiveness and local versus systemic effect. The highest possible class applies if a device can be classified according to several rules. 

**De Novo:** The De Novo process provides a pathway to classify novel medical devices for which general controls alone, or general and special controls, provide reasonable assurance of safety and effectiveness for the intended use, but for which there is no legally marketed predicate device. De Novo classification is a risk-based classification process. Devices that are classified into class I or class II through a De Novo classification request (De Novo request) may be marketed and used as predicates for future premarket notification [510(k)] submissions. [De Novo Classification request available at: https://www.fda.gov/medical-devices/premarket-submissions/de-novo-classification-request. Accessed October 3, 2020] 

**Directives:** Directives require EU countries to achieve a certain result, but leave them free to choose how to do so. EU countries must adopt measures to incorporate them into national law (transpose) in order to achieve the objectives set by the directive. National authorities must communicate these measures to the European Commission. Transposition into national law must take place by the deadline set when the directive is adopted (generally within 2 years). When a country does not transpose a directive, the Commission may initiate infringement proceedings. [Types of EU laws. Available at: https://ec.europa.eu/info/law/law-making-process/types-eu-law_en. Accessed on October 3, 2020]

**Double-reading**: AI software could potentially be used as a first reader, to screen examinations, with a radiologist reading only a subset of examinations selection based on the first reading. This approach however is currently not recommended [10]. However, the AI application could serve as a second reader in a variety of ways [39]. The AI software can be used as a second reader to provide input on a subset of cases selected by a radiologist. The AI software can also be used as a concurrent reader, where it serves as an aid to the radiologist. Finally, the AI software can be used for asynchronous quality control, with a human radiologist blind to the AI output, and discrepancies between the two arbitrated by a third radiologist or quality control committee.

**Ground Truth** or **Gold Standard:** Corresponds to what could be considered as the ideal result with respect to a given problem. The ground truth is synonymous with the gold standard when it refers to the diagnostic test or benchmark, which is best available under reasonable conditions. In the context of a clinical study, individual readers as well as an AI approach can then be compared to this benchmark in order to obtain performance metrics. It is important to remember that although it is denominated as truth, its quality varies greatly depending on its origin (e.g. biopsy, 2/3D imaging, longitudinal follow-up, etc.) and the individuals defining it (number, qualification, consensus criteria, etc.). An ill-defined ground truth can also introduce significant bias into a study.

**Intended use or purpose:** The intended use of a device, which is provided by the manufacturer in the intended use statement, is a description of what the device is supposed to be used for.

**Indications of use:** The indications of use, provided by the manufacturer in the indications of used statement, are the conditions or circumstances in which the device can be used.

**ISO27001:** ISO/IEC 27001:2013 specifies the requirements for establishing, implementing, maintaining and continually improving an information security management system within the context of the organization. It also includes requirements for the assessment and treatment of information security risks tailored to the needs of the organization. The requirements set out in ISO/IEC 27001:2013 are generic and are intended to be applicable to all organizations, regardless of type, size or nature.

*[ISO/IEC 27001:2013. Available at:* [*https://www.iso.org/standard/54534.html*](https://www.iso.org/standard/54534.html)*. Accessed on October 3, 2020]*

**ISO27017**: ISO/IEC 27017:2015 gives guidelines for information security controls applicable to the provision and use of cloud services by providing:

- additional implementation guidance for relevant controls specified in ISO/IEC 27002;
- additional controls with implementation guidance that specifically relate to cloud services.

This International Standard provides controls and implementation guidance for both cloud service providers and cloud service customers.

*[ISO/IEC 27017:2015. Available at:* [*https://www.iso.org/standard/43757.html*](https://www.iso.org/standard/43757.html)*. Accessed on October 3, 2020]*

**ISO27018:** ISO/IEC 27018:2014 establishes commonly accepted control objectives, controls and guidelines for implementing measures to protect Personally Identifiable Information (PII) for the public cloud computing environment.

In particular, ISO/IEC 27018:2014 specifies guidelines based on ISO/IEC 27002, taking into consideration the regulatory requirements for the protection of PII which might be applicable within the context of the information security risk environment(s) of a provider of public cloud services.

ISO/IEC 27018:2014 is applicable to all types and sizes of organizations, including public and private companies, government entities, and not-for-profit organizations, which provide information processing services as PII processors via cloud computing under contract to other organizations.

The guidelines in ISO/IEC 27018:2014 might also be relevant to organizations acting as PII controllers; however, PII controllers can be subject to additional PII protection legislation, regulations and obligations, not applying to PII processors. ISO/IEC 27018:2014 is not intended to cover such additional obligations.

*[ISO/IEC 27018:2014. Available at:* [*https://www.iso.org/standard/61498.html*](https://www.iso.org/standard/61498.html)*. Accessed on October 3, 2020]*

**Label:** In AI, a *label* refers to an annotation attached to an image or part of an image. This is typically generated by hand by radiologists, and can consist of a whole-image label (e.g., ‘COVID-19 patient’ or ‘healthy control’), rough region-of-interest or bounding box (e.g., ‘consolidation’ in chest X-ray), contours (e.g., in radiotherapy planning), or dense voxel-level annotations (e.g., anatomical segmentation for brain volumetry). It constitutes ‘ground truth’ and serves to train and evaluate AI applications.

**MDSAP:** The Medical Device Single Audit Program - a single audit of a medical device manufacturer’s Quality Management System (QMS), which satisfies the requirements of multiple regulatory jurisdictions.

**Notified Body**: A conformity assessment body designated in accordance with the Regulation or the Directive. A conformity assessment body is a body that performs third-party conformity assessment activities including calibration, testing, certification and inspection.

*[REGULATIONS REGULATION (EU) 2017/745 OF THE EUROPEAN PARLIAMENT AND OF THE COUNCIL of 5 April 2017 on medical devices. Available at:*

[*https://eur-lex.europa.eu/legal-content/EN/TXT/PDF/?uri=CELEX:32017R0745&from=EN.*](https://eur-lex.europa.eu/legal-content/EN/TXT/PDF/?uri=CELEX:32017R0745&from=EN.) *Accessed on September 30, 2020.]*

**Regulations**: Regulations are legal acts that apply automatically and uniformly to all EU countries as soon as they enter into force, without needing to be transposed into national law. They are binding in their entirety on all EU countries.

*[Types of EU laws. Available at:* [*https://ec.europa.eu/info/law/law-making-process/types-eu-law_en*](https://ec.europa.eu/info/law/law-making-process/types-eu-law_en)*. Accessed on October 3, 2020]*

**Software as a Medical Device (SaMD):** The term SaMD is defined by the International Medical Device Regulators Forum as software intended to be used for one or more medical purposes that perform these purposes without being part of a hardware medical device [7]. Of note, SaMD is capable of running on general purpose computing platforms. Also known as Medical Device Software (MDSW).

**Training:** An AI algorithm rests on a number of parameters (up to several millions for deep learning algorithms), that are estimated (‘learned’ or ‘trained’) from data. Training is an iterative procedure by which parameters are modified until the error of the model with respect to the training label(s) is minimized.

**Use case:** a specific clinical application for which the AI solution can be used.
